# Development of Biocompatible Ga_2_(HPO_4_)_3_ Nanoparticles as an Antimicrobial Agent with Improved Ga Resistance Development Profile against *Pseudomonas aeruginosa*

**DOI:** 10.3390/antibiotics12111578

**Published:** 2023-10-30

**Authors:** Huda Alamri, Guanyu Chen, Songping D. Huang

**Affiliations:** 1 Department of Chemistry and Biochemistry, Kent State University, Kent, OH 44240, USA; gchen10@kent.edu; 2 Department of Chemistry, College of Science, University of Jeddah, Jeddah 21589, Saudi Arabia

**Keywords:** Ga-based antimicrobial nanoparticles, antimicrobial resistance (AMR), top-down synthesis of nanomaterials, Ga-resistant bacterial phenotypes

## Abstract

Ga(III) can mimic Fe(III) in the biological system due to its similarities in charge and ionic radius to those of Fe(III) and can exhibit antimicrobial activity by disrupting the acquisition and metabolism of Fe in bacterial cells. For example, Ga(NO_3_)_3_ has been proven to be effective in treating chronic lung infections by *Pseudomonas aeruginosa* (*P. aeruginosa*) in cystic fibrosis patients in a recent phase II clinical trial. However, Ga(NO_3_)_3_ is an ionic compound that can hydrolyze to form insoluble hydroxides at physiological pH, which not only reduces its bioavailability but also causes potential renal toxicity when it is used as a systemic drug. Although complexion with suitable chelating agents has offered a varying degree of success in alleviating the hydrolysis of Ga(III), the use of nanotechnology to deliver this metallic ion should constitute an ultimate solution to all the above-mentioned problems. Thus far, the development of Ga-based nanomaterials as metalloantibiotics is an underexploited area of research. We have developed two different synthetic routes for the preparation of biocompatible Ga_2_(HPO_4_)_3_ NPs and shown that both the PVP- or PEG-coated Ga_2_(HPO_4_)_3_ NPs exhibit potent antimicrobial activity against *P. aeruginosa*. More importantly, such polymer-coated NPs do not show any sign of Ga-resistant phenotype development after 30 passes, in sharp contrast to Ga(NO_3_)_3,_ which can rapidly develop Ga-resistant phenotypes of *P. aeruginosa*, indicating the potential of using Ga_2_(HPO_4_)_3_ NPs a new antimicrobial agent in place of Ga(NO_3_)_3_.

## 1. Introduction

Antibacterial resistance has dramatically increased in the past several decades. This situation has led to an increased rate of morbidity and mortality caused by bacterial infections. Bacteria can rapidly evolve to develop new tactics to resist antibiotics and survive, which lowers the efficiency of existing antibiotics [1,2]. A recent report showed that more than one-third of clinical isolates of the Gram-negative bacterium *Pseudomonas aeruginosa* (*P. aeruginosa*) are now resistant to three or more antibiotic drugs [3]. The situation is very similar for other pathogenic organisms such as the Gram-positive *Staphylococcus aureus* (*S. aureus*), which is the cause of ~10% of hospital-acquired infections [4]. The number of infections caused by multidrug-resistant *S. aureus* (MRSA) has increased, with an increasing number of annual deaths related to MRSA infections [1]. Such problems are greatly worsened by the fact that the number of antibiotics approved for clinical use and in the drug development pipeline has dramatically declined over the past three decades [5]. There is an urgent need to develop new-generation antimicrobial agents with novel antimicrobial targets in order to win the battle against the rising antimicrobial resistance (AMR). Metalloantibiotics may offer a unique opportunity to hit the antimicrobial targets that are untouched by conventional antibiotics [6,7]. For example, targeting the acquisition and metabolism of iron by gallium has now been proven to be a viable approach to developing new metalloantibiotics to overcome AMR [8,9,10,11]. Due to the similarities of Ga(III) and Fe(III), including their identical ionic charge and comparable ionic radii (Ga^3+^ = 0.62 Å vs. Fe^3+^ = 0.65 Å for the high-spin electron configuration), Ga(III) can act as an Fe(III) mimic. However, Ga(III) cannot be reduced to Ga(II) under the physiological conditions to perform the redox reactions required of many enzymes and proteins that contain Fe as a cofactor. Consequently, the binding of Ga to Fe-containing enzymes and proteins will disrupt iron metabolism, thus affecting certain biological processes that are critical for bacterial survival and growth [12,13,14]. Recently, Ga(NO_3_)_3_, a clinical drug initially approved by the FDA for the treatment of hypocalcemia in cancer patients, has successfully completed a phase II clinical trial for the treatment of chronic lung infections by *P. aeruginosa* in cystic fibrosis patients [11]. However, as an ionic salt, there are several disadvantages of using Ga(NO_3_)_3_ as a systemic drug, including (i) the lack of hydrolytic stability at physiological pH as the Ga^3+^ ion can readily hydrolyze to form insoluble hydroxides to reduce its bioavailability; (ii) a relatively short half-life in the blood stream due to the high glomerular filtration rate of this small cation; and (iii) potential nephrotoxicity and cause of visual and hearing loss due to hydrolysis [15,16]. Although complexion with suitable chelating agents (e.g., maltol) has offered a varying degree of success in alleviating the hydrolysis of Ga(III) [17,18,19], the use of nanotechnology to deliver this metallic ion should constitute an ultimate solution to all the above-mentioned problems [20,21,22].

In this publication, we report on our efforts to develop biocompatible Ga-based nanoparticles (NPs) containing hydrogen phosphate to overcome the shortcomings of Ga(NO_3_)_3_. The potassium salt of hydrogen phosphate (i.e., dipotassium phosphate, or K_2_HPO_4_) is widely used as an emulsifier, stabilizer, texturizer and chelating agent in the food industry. For example, K_2_HPO_4_ is found in many imitation dairy creamers, dry powder beverages, mineral supplements and milk products, to prevent calcium from precipitating out, etc. The two main objectives of this study are (i) to explore the possibility of developing Ga_2_(HPO_4_)_3_ NPs as a broad-spectrum antimicrobial agent through their surface coating by a suitable polymer, as opposed to Ga(NO_3_)_3,_ which has been known to be only active against Gram-negative bacteria such as PA; and (ii) to investigate whether Ga_2_(HPO_4_)_3_ NPs would exhibit a delay in the development of Ga-resistant phenotypes of PA bacteria, as the rapid development of Ga-resistant phenotypes of PA bacteria treated with Ga(NO_3_)_3_ has become a genuine concern for its future clinical use. We show that the PVP- or PEG-coated Ga_2_(HPO_4_)_3_ NPs exhibit potent antimicrobial activity against *P. aeruginosa* but are rather ineffective against SA. More importantly, such coated NPs do not show any sign of Ga-resistant phenotype development after 30 passes. In sharp contrast, Ga-resistant phenotypes of PA bacteria treated with Ga(NO_3_)_3_ emerge rather rapidly in PA bacteria that are treated with Ga(NO_3_)_3_, i.e., after five passages. Overall, Ga_2_(HPO_4_)_3_ NPs still have desirable characteristics for use as a new antimicrobial agent against PA in place of Ga(NO_3_)_3_.

## 2. Results and Discussion

### 2.1. Determination of the Chemical Composition of the Reaction Product between Ga(NO_3_)_3_ and K_2_HPO_4_ Using the Conductimetric Titration

**Figure 1:**
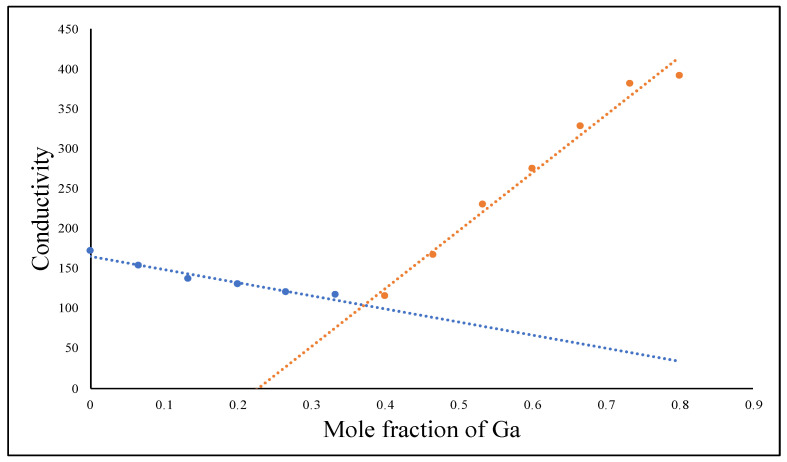
Curve of conductimetric titration measurements between Ga(NO_3_)_3_ and K_2_HPO_4_, indicating the formation of the solid product Ga_2_(HPO_4_)_3._ (the blue line represents the addition of Ga^3+^ ions, while the red line represents the addition of HPO_4_^2−^ ions).

Based on the law of definite proportions, when Ga(NO_3_)_3_ and K_2_HPO_4_ react with each other, only one distinct compound should form. Hence, we conducted a conductimetric titration experiment by reacting Ga(NO_3_)_3_ and K_2_HPO_4_ in aqueous solution to determine the stoichiometric ratio of the Ga^3+^ cation to the HPO_4_^2−^ anion in the Ga_m_(HPO_4_)_n_. In principle, the electrical conductivity of a solution depends on the number of free ions and the charge of these ions. When a solid product is formed, the total number of free ions will fall accordingly to indicate the equivalent point of a stochiometric ratio [23,24]. As shown in Figure 1, our conductimetric titration clearly showed a minimum conductivity point with a stoichiometric ratio of Ga^3+^: HPO_4_^2−^ of 2:3, indicating the chemical composition of the reaction product to be Ga_2_(HPO_4_)_3_. It is well-known that when Ga(NO_3_)_3_ and K_3_PO_4_ react with each other, the solid product formed has the chemical composition of GaPO_4_. The difference between Ga_2_(HPO_4_)_3_ and GaPO_4_—two solid-state Ga compounds of phosphates—is that Ga_2_(HPO_4_)_3_ NPs can be readily prepared using the simple coprecipitation method in aqueous solution, whereas GaPO_4_ NPs can only be synthesized using the hydrothermal method (vide infra).

### 2.2. Synthesis of PVP-Coated Ga_2_(HPO_4_)_3_ NPs Using the Coprecipitation Method

Through trial and error, we successfully synthesized stable Ga_2_(HPO_4_)_3_ NPs using the coprecipitation method in the presence of polyvinylpyrrolidone (PVP) as a surface stabilizer. Specifically, Ga_2_(HPO_4_)_3_ NPs were obtained by slowly adding an aqueous solution of Ga(NO_3_)_3_ (10 mM, 100 mL) containing 1 g of PVP (average MW = 8000) at the boiling point with another aqueous solution of K_2_HPO_4_ (30 mM, 100 mL) at the boiling point under vigorous stirring to afford a colorless colloidal solution with some turbidity. This solution immediately showed light scattering when a laser light was shone into it due to the Tyndall effect, indicating the formation of NPs in the solution (Figure S1). At this point, the pH of the resulting solution was about 6.9. After being stirred at 90 °C for 2 h and stored at room temperature overnight, the resultant NPs were dialyzed against distilled water using a cellulose tubular membrane to remove the byproducts and any unbound polymer. The solid product was collected after lyophilization. The Ga content of the powdered product was quantitatively analyzed using atomic absorption spectrometry (AAS). We noticed that the as-synthesized PVP-coated Ga_2_(HPO_4_)_3_ NPs were stable in solution for over six months.

### 2.3. Characterization of PVP-Coated Ga_2_(HPO_4_)_3_ NPs

**Figure 2:**
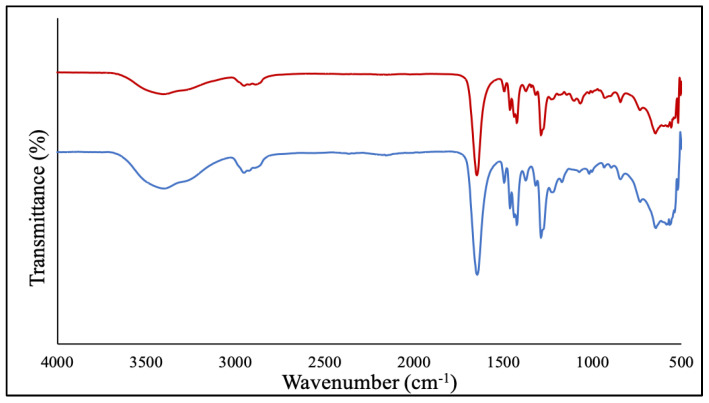
FT-IR spectrum of the PVP-coated Ga_2_(HPO_4_)_3_ NPs (red) in comparison with that of the neat PVP (blue).

The purified NPs were then characterized by Fourier transformed infrared spectroscopy (FT-IR), transmission electron microscopy (TEM) and energy-dispersive X-ray spectroscopy (EDS). First, the FT-IR spectrum of the PVP-coated Ga_2_(HPO_4_)_3_ NPs contained the major characteristic vibrations of the PVP polymer, confirming the presence of PVP attached to the surfaces of Ga_2_(HPO_4_)_3_ NPs (Figure 1). Note that the sample had been subjected to prolonged dialysis to remove the unbound PVP. In essence, the peak at 1645 cm^−1^ is attributable to the vibration of the carbonyl C=O group, while the peak at 2948 cm^−1^ is from the asymmetric stretching vibration of the CH_2_ backbone of PVP, and the peak at 1288 cm^−1^ is due to the vibration of the amide group in the heterocyclic ring of PVP as shown in Figure 2 [25,26].

**Figure 3:**
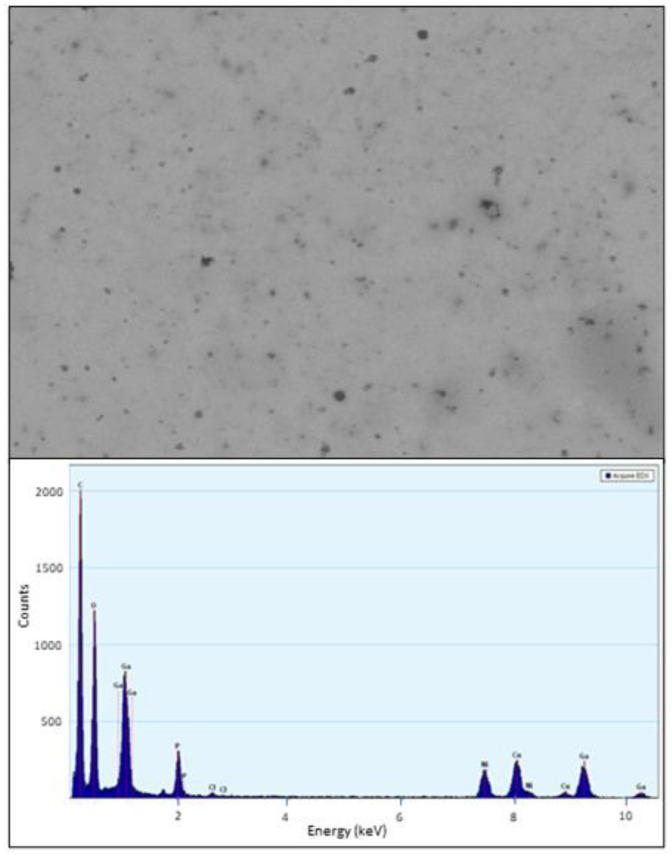
TEM images of PVP-coated Ga_2_(HPO_4_)_3_ NPs (**top**) and a representative EDS spectrum of PVP-coated Ga_2_(HPO_4_)_3_ NPs (**bottom**). Note: the signals of Cu and Ni are from the TEM grit.

The size and morphology of PVP-coated Ga_2_(HPO_4_)_3_ NPs were determined by TEM. The TEM images show that the NPs have an ill-defined semi-spherical shape, having a size ranging from 10 to 20 nm, with poor crystallinity (Figure 3). Additionally, the composition analysis by energy dispersive spectroscopy (EDS) from different selected areas of several NPs showed distinctive signals of elements Ga, P and O (Figure 3). 

### 2.4. Investigation of Antibacterial Activity of PVP-Coated Ga_2_(HPO_4_)_3_ NPs

**Figure 4:**
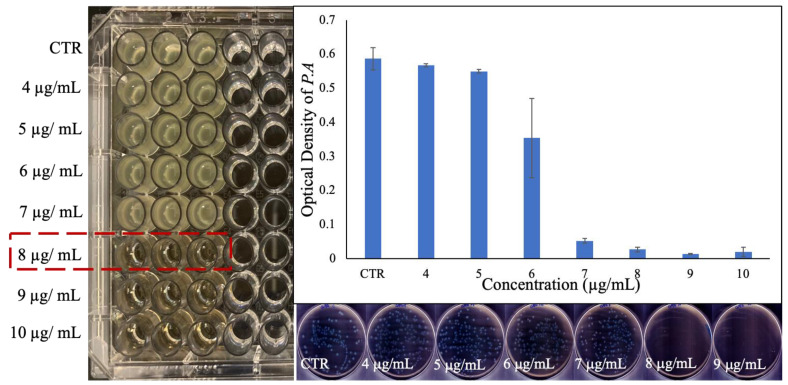
Results of the MIC determination for PVP-coated Ga_2_(HPO_4_)_3_ NPs against *P. aeruginosa* using the broth dilution technique in the well-plate (**left**), the measured optical density values of the bacteria treated with various concentrations of NPs (**top right**), and the agar plates of the bacteria treated with different concentrations of NPs (**down right**).

The minimum inhibitory concentration (MIC) of the PVP-coated Ga_2_(HPO_4_)_3_ NPs against the Gram-negative *Pseudomonas aeruginosa* bacteria (ATCC 15692) was found to be 8 µg/mL (Figure 4). The optical density measurement obtained from the absorbance at 600 nm also confirmed the growth inhibition of *P. aeruginosa* by such NPs (Figure 4). A significant decrease in the optical densities was observed at around 8 µg/mL (i.e., 1 × MIC), and the higher concentrations, in sharp contrast to the control of untreated bacteria, suggest that PVP-coated Ga_2_(HPO_4_)_3_ NPs may be a potent antimicrobial agent against *P. aeruginosa*. It should be noted that the MIC of Ga(NO_3_)_3_ measured by us under similar conditions using the same culture medium was found to be 12 µg/mL, while this value decreased to 8 µg/mL when sodium citrate was added to the medium [27]. To further investigate the quantitative aspect of the antibacterial efficacy of the PVP-coated Ga_2_(HPO_4_)_3_ NPs, both optical density values were measured, and colony-forming units (CFUs) were enumerated by plating 50 µL of each bacterial cultured treated with a specific concentration and incubation for 18 h. As shown in Figure 4, no bacterial colonies grew in the plates that had been inoculated with bacteria treated with the MIC value (8 µg/mL) or higher concentrations, confirming that the PVP-coated Ga_2_(HPO_4_)_3_ NPs are indeed a potent antimicrobial agent against *P. aeruginosa*. 

**Figure 5:**
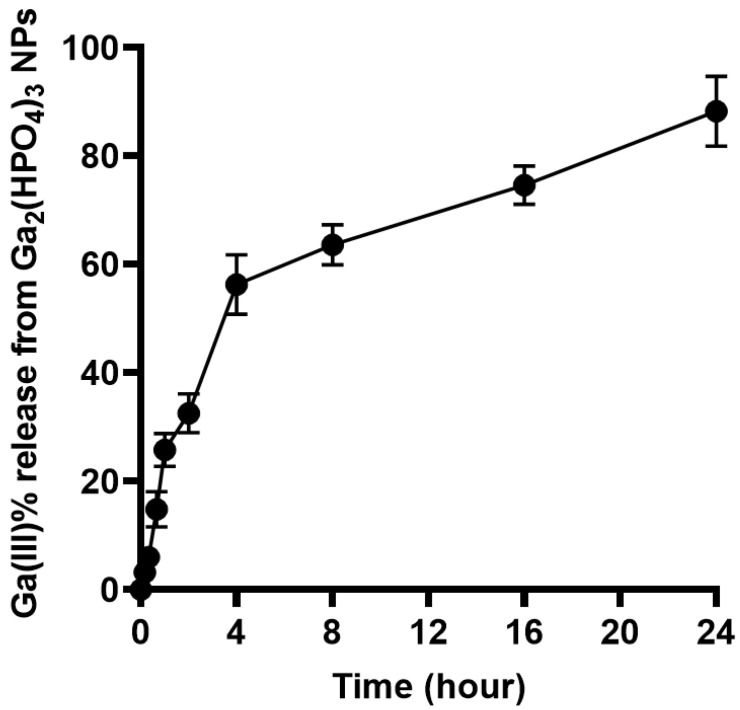
Percent Ga(III) release from PVP-coated Ga_2_(HPO_4_)_3_ NPs versus time (hour).

To evaluate the kinetics of the Ga^3+^ release from such NPs, a volume of 10 mL of PVP-coated Ga_2_(HPO_4_)_3_ NPs was sealed in a dialysis bag (MWCO = 12,000–14,000). The latter was submerged in 40 mL of citrate buffer solution with pH = 3.0 in a 55 mL test tube. The test tube was then placed in a shaker set at 37 °C with a speed of 180 rpm. During the experiment, at predetermined intervals of 10, 20, 40, 60, 120, 240, 480, 960 and 1440 min, an aliquot of 2 mL solution was removed and treated with a drop of 70% concentrated nitric acid in preparation for gallium ion concentration analysis using atomic absorption spectrometry (AAS). All experiments were conducted in triplicate to ensure reproducibility and consistency in the results. As shown in Figure 5, the Ga(III) release underwent two different phases. First, within 4 h, the Ga(III) release rapidly reached the 60% level. Second, the remaining phase of Ga(III) release was considerably slower, i.e., it took 20 h for the Ga(III) release to reach from the 60% to the 100% level. 

Next, we investigated the antibacterial activity of PVP-coated Ga_2_(HPO_4_)_3_ NPs against the Gram-positive *Staphylococcus aureus* bacteria (ATCC 6538) using the same experimental techniques as the above studies. However, our results showed no visible inhibition of bacterial growth at any of the concentrations we tested, including the highest possible concentration of 48 µg/mL (Figure S2). The measured optical density also showed no statistically significant decrease in the absorbance of 600 nm that was observed in this concentration range (Figure S2). Finally, the enumeration of CFUs obtained from the agar plates also failed to show any discernible inhibitory effect. Together, these results indicate that PVP-coated Ga_2_(HPO_4_)_3_ NPs do not exhibit any significant antibacterial activity against the Gram-positive bacteria *S. aureus*, which becomes an obstacle to developing these NPs as a broad-spectrum antimicrobial agent. 

It should be noted that the major difference between Gram-positive and Gram-negative bacteria is the thickness of the peptidoglycan layer and the existence or absence of an outer cell membrane in their cell wall. Compared to that of Gram-negative bacteria, the cell wall of Gram-positive bacteria has a thick multilayer of peptidoglycan outside the cell membrane [28]. We then speculated that the change of the surface-coating polymer from PVP to polyethylene glycol (PEG) might improve the ability of such NPs to penetrate the cell wall of Gram-positive bacteria. However, we were unable to prepare stable PEG-coated Ga_2_(HPO_4_)_3_ NPs using the same coprecipitation method with PEG in place of PVP. We subsequently developed a top-down synthetic route to produce PEG-coated Ga_2_(HPO_4_)_3_ NPs from the bulk samples using the sonication technique. 

### 2.5. Synthesis of the Bulk Ga_2_(HPO_4_)_3_ Sample

The bulk Ga_2_(HPO_4_)_3_ sample was synthesized under the same conditions as described above for the synthesis of PVP-coaled Ga_2_(HPO_4_)_3_ NPs except that no surface-coating polymers were used. Specifically, the Ga_2_(HPO_4_)_3_ precipitate was obtained by directly mixing an aqueous solution of Ga(NO_3_)_3_ (100 mM, 50 mL) at the boiling point with another aqueous solution of excess K_2_HPO_4_ (300 mM, 50 mL) at the boiling point. The mixing resulted in the immediate formation of a white precipitate of Ga_2_(HPO_4_)_3_. The product was filtered, washed thoroughly with deionized water to remove any unreacted salts and dried at 180 °C for 4 h (Figure S3). 

### 2.6. Synthesis of the PEG-Coated Ga_2_(HPO_4_)_3_ NPs Using the Top-Down Sonication Method

The PEG-coated Ga_2_(HPO_4_)_3_ NPs were produced by sonicating the bulk sample of K_2_HPO_4_ using DMSO the solvent and PEG (average MW = 8000) as the surface-coating agent, followed by sonication for 48 h to result in a translucent dispersion that also showed light scattering when a laser light was shone into it, confirming that size reduction of the particles occurred to produce NPs. The Ga content of these PEG-coated Ga_2_(HPO_4_)_3_ NPs was determined using AAS. 

### 2.7. Characterization of PEG-Coated Ga_2_(HPO_4_)_3_ NPs

**Figure 6:**
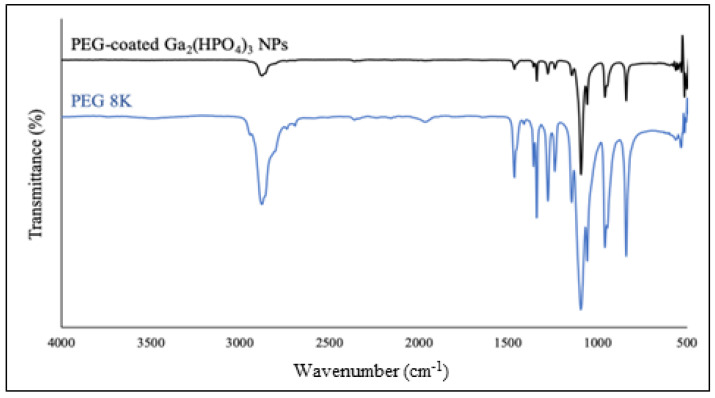
FT-IR spectrum of the PEG-coated Ga_2_(HPO_4_)_3_ NPs (black) in comparison with that of the neat PVP (blue).

After purification by membrane dialysis, collection of the solid product was performed by lyophilization. Such NPs were first analyzed by FT-IR spectroscopy. As shown in Figure 6, the PEG-coated NPs contain all the major absorption bands of PEG, confirming that the polymer is bound to the surfaces of the NPs. Specifically, the characteristic absorption band at 2884 cm^−1^ is attributable to the vibration of stretching C–H. The observed bands at 1467 cm^−1^ and 1342 cm^−1^ are due to the C–H bending vibrations. The peaks at 1279 cm^−1^ and 1094 cm^−1^ are assigned to C–O–H [29]. 

**Figure 7:**
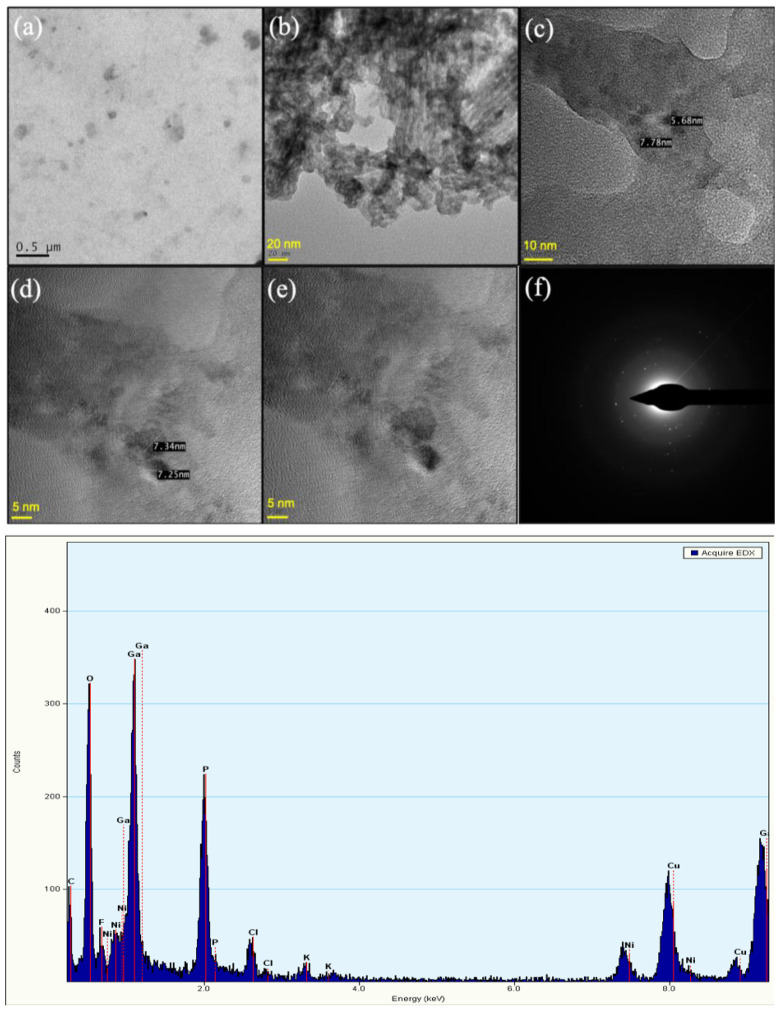
TEM images of PEG-coated Ga_2_(HPO_4_)_3_ NPs with different magnifications (**top** (**a**–**e**)), the HRTEM image of single Ga_2_(HPO_4_)_3_ NPs, showing the single crystal nature of such NPs (**top** (**f**)), and a representative EDS spectrum of PEG-coated Ga_2_(HPO_4_)_3_ NPs (**bottom**). Note: the signals of Cu and Ni are from the TEM grit.

The TEM images show that the PEG-coated Ga_2_(HPO_4_)_3_ NPs have quasi-spherical shapes with sizes <10 nm (Figure 7a–e) and a relatively uniform shape and size distribution. At high magnification of the TEM, the images reveal a crystallinity of the Ga_2_(HPO_4_)_3_ NPs. Moreover, the selected area electron diffraction (SAED) pattern consists of distinct diffraction points, indicating that such NPs are single crystallites (Figure 7f) [30]. On the other hand, the EDS measurements show distinctive signals of Ga, P and O, as shown in Figure 7. 

### 2.8. Investigation of Antibacterial Activity of PEG-coated Ga_2_(HPO_4_)_3_ NPs

**Figure 8:**
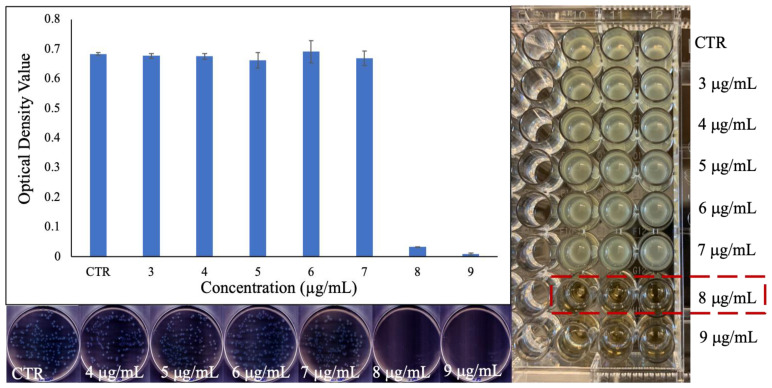
Results of the MIC determination for PEG-coated Ga_2_(HPO_4_)_3_ NPs against *P. aeruginosa* using the broth dilution technique in the well-plate (**right**), the measured optical density values of the bacteria treated with various concentrations of NPs (**top left**), and the agar plates of the bacteria treated with different concentrations of NPs (**down left**).

The MIC of the PEG-coated Ga_2_(HPO_4_)_3_ NPs against the same strain of *P. aeruginosa* bacteria (i.e., ATCC 15692) that was used to determine the antimicrobial activity of the PVP-coated Ga_2_(HPO_4_)_3_ NPs in the above was found to be 8 µg/mL (Figure 8)—an identical value as that of PVP-coated Ga_2_(HPO_4_)_3_, indicating that the change of surface-coating polymer from PVP to PEG does not have any discernible effect on the antimicrobial activity of Ga_2_(HPO_4_)_3_ NPs against this Gram-negative strain of bacteria. This observation is also confirmed by measurements of both optical density values and CFUs (Figure 8).

Again, we investigated the antibacterial activity of PEG-coated Ga_2_(HPO_4_)_3_ NPs against *S. aureus* bacteria (ATCC 6538) in the hope that the PEG surface coating would improve the interactions of these NPs with the thick multilayer of peptidoglycan interwoven with teichoic acid chains on the cell wall of Gram-positive bacteria. Unfortunately, all our experimental results, including the MIC determination, optical density measurements and CFU enumerations show that PEG-coated Ga_2_(HPO_4_)_3_ NPs are not active against *S. aureus* bacteria (see Figure S4). 

### 2.9. Investigation of Ga Resistance Development of PEG-Coated Ga_2_(HPO_4_)_3_ NPs

**Figure 9:**
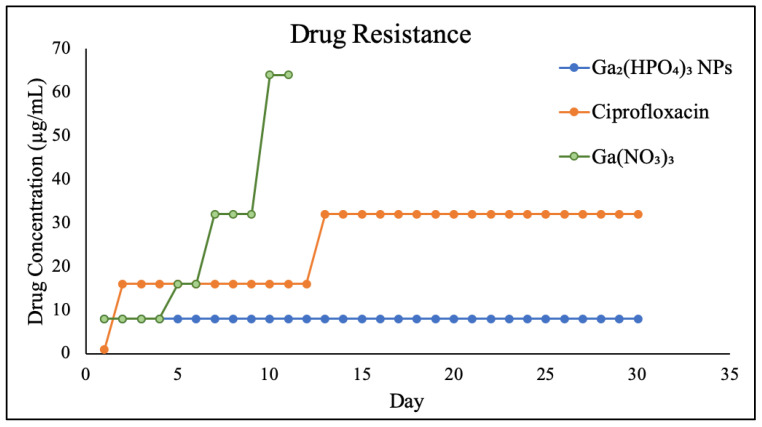
Results of Ga resistance development assays of PEG-coated Ga_2_(HPO_4_)_3_ in comparison with Ga(NO_3_)_3_ and ciprofloxacin.

The Ga resistance development of PEG-coated Ga_2_(HPO_4_)_3_ NPs, in comparison with Ga(NO_3_)_3_ and ciprofloxacin, was examined in *P. aeruginosa* for a period of 30 days. The results show that the wild-type *P. aeruginosa* bacteria remain susceptible to PEG-coated Ga_2_(HPO_4_)_3_ NPs after repetitive exposures to such NPs at the sub-lethal dose for 30 days, showing a remarkable reluctance to develop any Ga-resistant phenotypes. In sharp contrast, both Ga(NO_3_)_3_ and ciprofloxacin—one of the most frequently prescribed antibiotics in the clinical treatment of bacterial infections—exhibited a rapid development of drug resistance, attesting to the different antimicrobial mode of action by PEG-coated Ga_2_(HPO_4_)_3_ vs. those of Ga(NO_3_)_3_ and ciprofloxacin (Figure 9). 

## 3. Conclusions

In conclusion, biocompatible Ga_2_(HPO_4_)_3_ NPs that are surface functionalized with PVP or PEG can be readily prepared using either a direct coprecipitation method in aqueous solution or a top-down sonication technique. Both the PVP- and PEG-coated Ga_2_(HPO_4_)_3_ NPs exhibit potent antimicrobial activity that is comparable to Ga(NO_3_)_3_ against *P. aeruginosa*. Similarly to Ga(NO_3_)_3_, these NPs remain ineffective against *S. aureus*. Nevertheless, our findings in this study strongly suggest that Ga-based NPs may represent a feasible solution to the various problems and shortcomings of molecular Ga compounds in the battle against AMR.

## 4. Materials and Methods

### 4.1. Chemical Reagents and Biological Materials

All chemical reagents were obtained from commercial sources and used without further purification. Gallium nitrate hydrate (Ga(NO_3_)_3_∙*x*H_2_O), dipotassium phosphate (K_2_HPO_4_), polyvinylpyrrolidone (PVP; average MW = 8000), polyethylene glycol (PEG; average MW = 8000), dimethyl sulfoxide (DMSO), gallium standard (1000 ± 10 µg/mL) were all purchased from Sigma-Aldrich. Bacterial strains, growth media and antibiotics, Gram-positive bacteria (ATCC 6538) and Gram-negative bacteria (ATCC 15692) were purchased from American Type Culture Collection. Tryptic broth powder (TSB), tryptic soy agar (TSA), nutrient broth (NB) and nutrient agar (NA) were purchased from Fisher Scientific.

### 4.2. Conductimetric Measurements to Determine the Stoichiometry

Solution conductivity measurements were employed to determine the stoichiometric ratio of a precipitate formed between Ga^3+^ and HPO_4_^2−^. A solution of 1.0 mM K_2_HPO_4_ was titrated with a 1.0 mM solution of Ga(NO_3_)_3_ at 25 °C forming a white precipitate of Ga_2_(HPO_4_)_3_ using an OAKTON PC 700 pH/Conductivity meter. The stochiometric composition was determine by electrical conductivity in micro-siemens (μS) measured in 12 different titrations with different molar ratios of both reactants in each titration. The results of electrical conductivity readings were plotted against the mole ratio of Ga^3+^ ions to determine the stoichiometric ratio of Ga^3+^ and HPO_4_^2−^. 

### 4.3. Synthesis of PVP-Coated Ga_2_(HPO_4_)_3_ NPs

A 100 mL aqueous solution containing 0.418 g of Ga(NO_3_)_3_ and 1 g of PVP (average MW = 8000) was heated to its boiling point and stirred at this temperature for 15 min. Separately, a 100 mL aqueous solution of 1.3694 g of K_2_HPO_4_ was also heated to its boiling point. The gallium nitrate/polymer solution was added slowly into the dipotassium phosphate solution under vigorous stirring to give a colorless colloidal solution with some turbidity. After being heated on a hot plate at 90 °C for 2 h and stirred at room temperature overnight, the NP solution was dialyzed against distilled water using cellulose tubular membrane (MWCO = 12,000–14,000) to remove the byproducts and any unbound polymer. The PVP-coated Ga_2_(HPO_4_)_3_ NPs were collected as a powdered product after lyophilization. The Ga content of the powdered product was quantitatively analyzed using atomic absorption spectrometry (AAS). 

### 4.4. Synthesis of PEG-Coated Ga_2_(HPO_4_)_3_ NPs

1 mg of the bulk Ga_2_(HPO_4_)_3_ was added to 1 mL of DMSO in the presence of 100 mg of PEG (average MW = 8000) and sonicated for 48 h. The resulting suspension was clear and showed light scattering when a laser light was shone to it, indicating the formation of NPs. The PEG-coated Ga_2_(HPO_4_)_3_ NPs were collected as a powdered product after lyophilization. The Ga content of the powdered product was quantitatively analyzed using atomic absorption spectrometry (AAS). 

### 4.5. TEM and HRTEM Imaging Studies

The NPs were first dispersed in ethanol (95%) and sonicated for 30 min. Next, one drop of NP suspension was placed onto a carbon-coated copper TEM grid (400 mesh) and allowed to air-dry before the analysis. The TEM specimens were imaged using an FEI Tecnai F20 Transmission Electron Microscope equipped with a field emission gun and analyzed at 200 KV. The energy dispersive X-ray spectroscopic (EDX) data were acquired for a selected area of the sample with the integrated scanning TEM (STEM) unit and attached EDAX spectrometer. The spatial resolution is <1 nm through the acquisition of high resolution with high-angle angular dark field (HAADF) images, which are sensitive to atomic number (Z) contrast. 

### 4.6. Antibacterial Activity Assays

The bacteria were cultured in tryptic soy broth (TSB) for Bacterial *S. aureus* SA (ATCC 6538) and nutrient broth (NB) for *P. aeruginosa* (ATCC 15692), respectively. The bacterial suspension was prepared by transferring an isolated colony from a streak plate of tested bacterial strain into 5 mL of TSB media, followed by incubation at 37 °C with shaking at 180 rpm for 18 h. Next, 50 μL was sub-cultured in 5 mL fresh TSB media and incubated for additional 4 h. The cell density of bacterial suspension after incubation was 10^9^ CFU/mL. Tested bacterial cell concentrations for studies were adjusted to have a final concentration of bacteria in the tested suspension to be at 10^6^ CFU/mL. 

### 4.7. MIC Assays

The bacterial suspension cultured overnight was adjusted to the targeted bacterial concentration of 10^6^ CFU/mL. Next, drugs to be tested were added to the bacterial suspension, and the final concentration of DMSO in the bacterial suspension is 0.2% in 96-well plates. Following the treatment with drugs, bacteria were incubated for 18 h at 37 °C and shaking speed 180 rpm. The MIC was determined as the lowest concentration that shows no visible bacterial growth with unaided eyes.

### 4.8. Colony Forming Unit (CFU/mL) Assays

Bacteria grown in the cell culture medium without NPs were used as the control in all such experiments. After 18 h of incubation, a diluted mixture containing the appropriate amount of NPs was spread across the agar plate using a glass spreader. The colonies found in each plate were then counted and converted to numbers of CFU/mL. Triplicates were obtained for all technical and biological parameters. 

### 4.9. Drug Resistance Development Assays

Typically, in 96-well plates, serial twofold dilutions of tested drugs with a bacterial strain were prepared to have the targeted bacterial cell concentration of 10^6^ CFU/mL. The bacterial suspensions were added in triplicate to 96-well plates and incubated overnight in an incubator at 37 °C with shaking speed at 180 rpm. A bacterial suspension without drug was used as a negative control. After 18 h of incubation, the MIC was determined by measuring the O.D. value using a microplate reader in which the lowest concentration of the drug that has an O.D. reading similar to the control is the MIC. Next, 10 µL of the sub-MIC was incubated with the tested drug and incubated to determine the MIC. This procedure was repeated for 30 days to test the potential ability of tested bacteria to develop resistance to the drug. A SpectraMax M4 Spectrophotometer was used to measure absorbance in the 96-well plates. 

## Data Availability

All data generated or analyzed during this study are included in this published article and its Supplementary Information Files.

## Supplementary Materials

The following supporting information can be downloaded at: https://www.mdpi.com/article/10.3390/antibiotics12111578/s1, All data generated or analyzed during this study are included in this published article and its supplementary information files are available free of charge. Figure S1: Schematic of the synthesis of PVP-coated Ga2(HPO4)3 NPs with the Tyndall effect exhibited by the product. Figure S2: Results of the MIC determination for PVP-coated Ga2(HPO4)3 NPs against S. aureus using the broth dilution technique in the well-plate (left) and the measured optical density values of the bacteria treated with various concentrations of NPs. Figure S3: Schematic of the two-step synthesis of PEG-coated Ga2(HPO4)3 NPs with the Tyndall effect exhibited by the product: (A) the preparation of the bulk sample and (B) the synthesis of PEG-coated Ga2(HPO4)3 NPs. Figure S4: Results of the MIC determination for PEG-coated Ga2(HPO4)3 NPs against S. aureus using the broth dilution technique in the well-plate (left) and the measured optical density values of the bacteria treated with various concentrations of NPs.
