# Supplementary material for: Development of Biocompatible Ga2(HPO4)3 Nanoparticles as an Antimicrobial Agent with Improved Ga Resistance Development Profile against Pseudomonas aeruginosa
Source: Antibiotics (Basel). 2023 Oct 30;12(11):1578. doi: 10.3390/antibiotics12111578 (PMC10668710; doi:10.3390/antibiotics12111578)
Supplement: Supplementary file 1 [file antibiotics-12-01578-s001.zip › antibiotics-2510225-supplementary.pdf]

Supplementary Information

Article

Development of Biocompatible $Ga_2(HPO_4)_3$ Nanoparticles as an Antimicrobial Agent with Improved Ga Resistance Development Profile against *Pseudomonas aeruginosa*

Huda Alamri [1,2,*], Guanyu Chen [1] and Songping D. Huang [1,*]

[1] Department of Chemistry and Biochemistry, Kent State University, Kent, OH 44240, USA; gchen10@kent.edu
[2] Department of Chemistry, College of Science, University of Jeddah, Jeddah 21589, Saudi Arabia
* Correspondence: halamri@uj.edu.sa (H.A.); shuang1@kent.edu (S.D.H.)

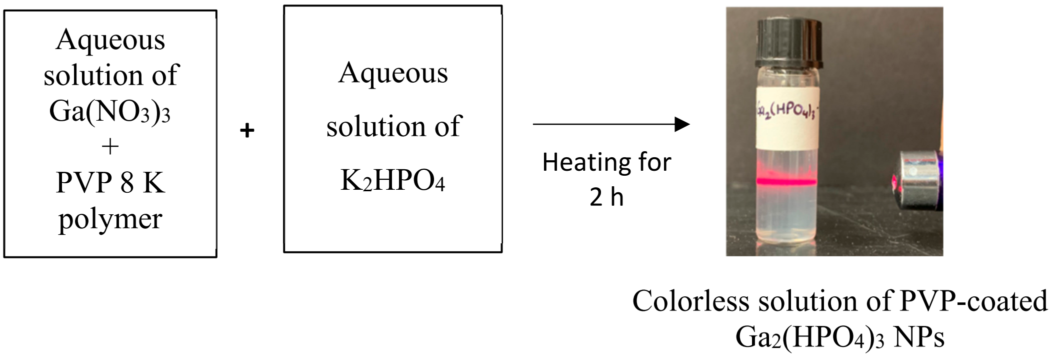

Figure S1. Schematic of the synthesis of PVP-coated $Ga_2(HPO_4)_3$ NPs with the Tyndall effect exhibited by the product.

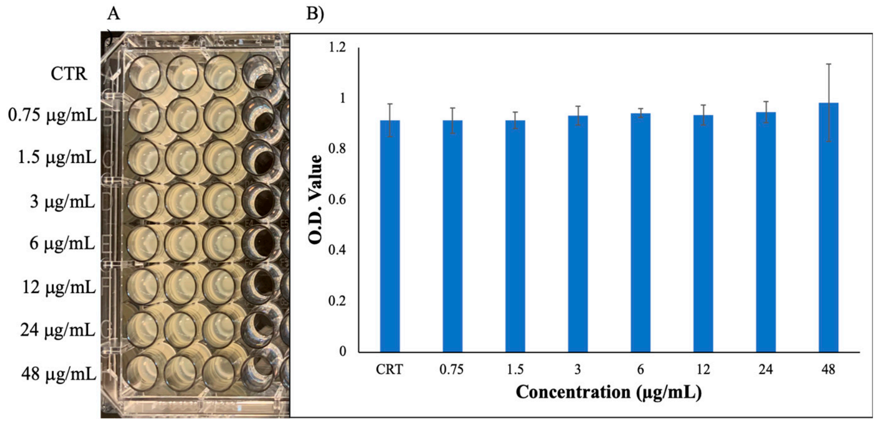

Figure S2. Results of the MIC determination for PVP-coated $Ga_2(HPO_4)_3$ NPs against *S. aureus* using the broth dilution technique in the well-plate (left) and the measured optical density values of the bacteria treated with various concentrations of NPs.

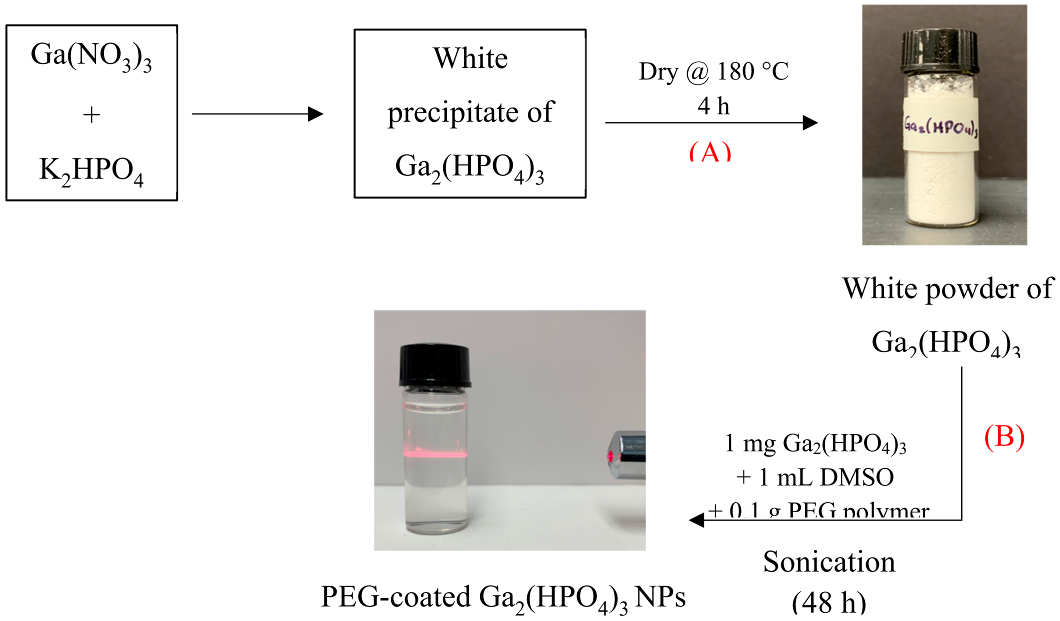

Figure S3 Schematic of the two-step synthesis of PEG-coated $Ga_2(HPO_4)_3$ NPs with the Tyndall effect exhibited by the product: (A) the preparation of the bulk sample and (B) the synthesis of PEG-coated $Ga_2(HPO_4)_3$ NPs.

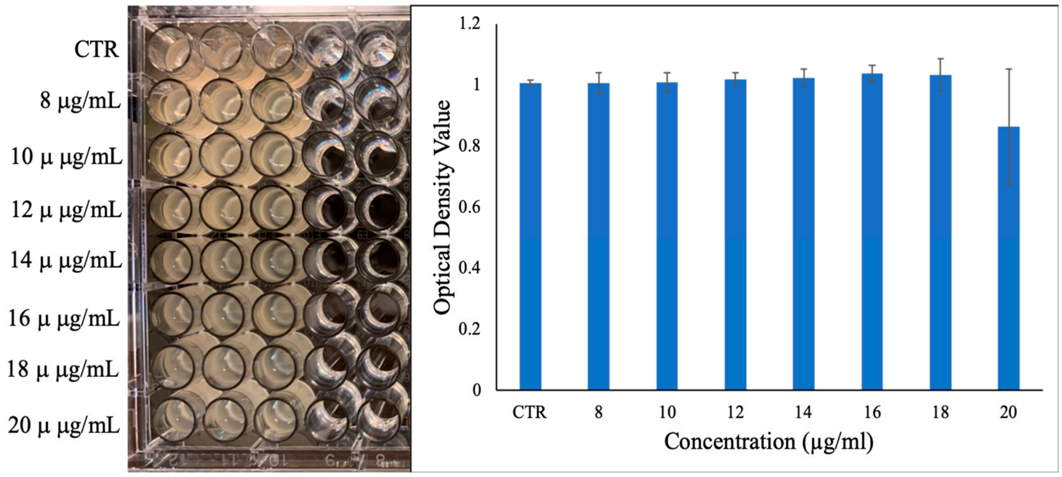

Figure S4. Results of the MIC determination for PEG-coated $Ga_2(HPO_4)_3$ NPs against *S. aureus* using the broth dilution technique in the well-plate (left) and the measured optical density values of the bacteria treated with various concentrations of NPs.
